# Encapsulation of HRP-Immobilized Silica Particles into Hollow-Type Spherical Bacterial Cellulose Gel: A Novel Approach for Enzyme Reactions within Cellulose Gel Capsules

**DOI:** 10.3390/gels10080516

**Published:** 2024-08-06

**Authors:** Toru Hoshi, Masashige Suzuki, Takao Aoyagi

**Affiliations:** 1 Department of Materials and Applied Chemistry, College of Science and Technology, Nihon University, 1-8-14, Kanda-Surugadai, Chiyoda-ku, Tokyo 101-8308, Japan; aoyagi.takao@nihon-u.ac.jp; 2 Department of Materials and Applied Chemistry, Graduate School of Science and Technology, Nihon University, 1-8-14, Kanda-Surugadai, Chiyoda-ku, Tokyo 101-8308, Japan; csma19025@g.nihon-u.ac.jp

**Keywords:** hollow-type spherical bacterial cellulose gel, encapsulation, enzyme immobilization, release behavior

## Abstract

We revealed that the encapsulation of enzyme-immobilized silica particles in hollow-type spherical bacterial cellulose (HSBC) gels enables the use of the inside of HSBC gels as a reaction field. The encapsulation of horseradish peroxidase (HRP)-immobilized silica particles (Si-HRPs, particle size: 40–50 μm) within HSBC gels was performed by using a BC gelatinous membrane produced at the interface between *Komagataeibacter xylinus* suspension attached onto an alginate gel containing Si-HRPs and silicone oil. After the biosynthesis of the BC gelatinous membrane, formed from cellulose nanofiber networks, the alginate gel was removed via immersion in a phosphate-buffered solution. Si-HRP encapsulated HSBC gels were reproducibly produced using our method with a yield of over 90%. The pore size of the network structure of the BC gelatinous membrane was less than 1 μm, which is significantly smaller than the encapsulated Si-HRPs. Consequently, the encapsulated Si-HRPs could neither pass through the BC gelatinous membrane nor leak from the interior cavity of the HSBC gel. The activity of the encapsulated HRPs was detected using the 3,3′,5,5′-tetramethylbenzidine (TMB)-H_2_O_2_ system, demonstrating that this method can encapsulate the enzyme without inactivation. Since HSBC gels are composed of a network structure of biocompatible cellulose nanofibers, immune cells cannot enter the hollow interior, thus, the enzyme-immobilized particles encapsulated inside the HSBC gel are protected from immune-cell attacks. The encapsulation technique demonstrated in this study is expected to facilitate the delivery of enzymes and catalysts that are not originally present in the in vivo environment.

## 1. Introduction

Enzymes, as biocatalysts, provide clean, environmentally friendly, and specific methods for biochemical reactions under mild conditions [1,2,3,4]. However, the use of enzymes is limited due to their high cost and low reusability. Moreover, the absence of an appropriate mechanism to protect the enzyme from protease attack, which occurs in almost all biological systems, is a major obstacle to achieving optimal activity [5]. Furthermore, the low operational stability of enzymes during biochemical reactions is also a challenge. Enzyme immobilization is an effective way to overcome the aforementioned issues. Immobilization methods include the inclusion and encapsulation of enzymes into matrices, or binding them to various surfaces [6,7]. Among various chemical catalysts, hydrogels can be used as effective carriers for immobilizing enzymes. An ideal hydrogel matrix should possess the following properties: (a) cost-effectiveness, (b) inertness, (c) stability, (d) excellent mechanical strength, (e) a lack of effect on the enzyme reaction products, and (f) the prevention of nonspecific adsorption and bacterial contamination. Hydrogels are superior to other immobilization materials because of their high water content, which can reduce the denaturation of enzymes and help maintain their activity [8]. Bialal et al. reported that horseradish peroxidase (HRP) immobilized in an agarose–chitosan hydrogel (ACH) maintained stability across a wide range of pH and temperatures [9]. Additionally, this immobilized enzyme remained reusable with a minimal loss of activity. The hydrophilic and highly porous polymer network of hydrogels makes them suitable for encapsulation [5]. However, one disadvantage of encapsulation is enzyme leakage during storage in an aqueous solution [10]. Naghdi et al. reported that laccase encapsulated in a chitosan–biochar composite leaked 2% over a 5-day period [11].

Enzyme and cell encapsulation technologies have been applied in diverse fields, such as pharmaceuticals, food sciences, paints, cosmetics, and adhesives. In particular, encapsulation of islet cells with alginate has proven effective in treating type I diabetes [12,13], though biocompatibility remains a concern. Consequently, alternative microencapsulated materials with alginate, including polyethylene glycol, poly(methyl methacrylate), agarose, chitosan, collagen, and gelatin are being explored [14,15,16].

Numerous natural and synthetic polymers have been examined to identify the optimal encapsulating material. In addition, several methods of fabricating capsules have also been developed, including chemical methods, such as interfacial polymerization and in situ polymerization (suspension, emulsion, and dispersion polymerization), physicochemical methods, such as coacervation, layer-by-layer assembly, and sol–gel encapsulation, and physicomechanical methods, such as spray-drying, co-extrusion, and phase-inversion precipitation [17]. Important characteristics of capsules such as membrane permeability, mechanical stability, and adhesion properties are influenced by material, size, and morphology. Capsules often have nanoscale pore sizes but can easily collapse under high osmotic pressure because of limited solute exchange with the bulk environment [18,19]. However, more open microcapsule structures can encapsulate fragile cellular cargo while allowing nutrient transport [20]. As a result, resistance to stretch and rupture is often limited, as materials such as poly(ethylene glycol) (PEG) and 2-hydroxyethyl acrylate (HEA) hydrogels can be too soft [21,22] to resist shearing and tearing. A significant advancement would be the fabrication of materials with substantial tensile strength and a flexible response to deformation, which can be achieved through a novel microstructure.

One material with useful hierarchical mechanical and structural properties is bacterial cellulose (BC). BC can be readily produced via the fermentation of bacteria such as *Acetobacter Xylinum*, and used as a hydrogel that offers strong mechanical and physical properties, including a high tensile strength, modulus, water-holding capacity, porosity, crystallinity, and good biocompatibility [23]. Controlling the shape of BC gel during production is difficult, thus, a new method to facilitate the production of BC gels with desired shapes suitable for specific purposes is required [24,25,26]. Recent reports have demonstrated the successful preparation of BC capsules using a BC gelatinous membrane biosynthesized by bacteria at the oil–water interface [20,27]. The BC gelatinous membrane, which comprises a network structure of cellulose nanofibers, creates a highly porous structure that allows for the exchange of various molecules smaller than the pore size of the cellulose nanofiber network.

On the other hand, the gelatinous membrane of BC capsules acts as a barrier against larger particles. In our previous work, we developed an encapsulation method using BC [28], in which a BC gelatinous membrane was generated at the interface between silicone oil and a cell suspension attached to the surface of a spherical alginate gel containing the target substance. Then, the alginate gel was dissolved to obtain a substance-encapsulated, hollow-type, spherical BC (HSBC) gel. This encapsulation with HSBC gel by the particle-preloaded droplet cultivation method using alginate gel can encapsulate particles regardless of whether they are organic or inorganic substances. The activated carbon encapsulated via this method did not leak externally from the interior cavity of the HSBC gel. The HSBC gel also did not suppress the mass transfer of solute molecules; the encapsulated activated carbon effectively adsorbed indole, a precursor of the uremic causative agent [29]. Additionally, the activated carbon encapsulated in the HSBC gel remained stable under acidic and basic conditions. By maintaining its structural integrity, the activated carbon encapsulated in the HSBC gel is expected to traverse the gastrointestinal tract without leaking. Due to this stability, activated carbon-encapsulated HSBC gel is being investigated as a new formulation for treating renal failure and enhancing patient compliance with medication regimens [30].

In this study, we revealed that the encapsulation of enzyme-immobilized silica particles using HSBC gels enables the use of the inside of HSBC gels as a reaction field. To prevent enzyme leakage, the enzyme (horseradish peroxidase (HRP)) was immobilized via glutaraldehyde on amino-modified silica particles larger than the pore size of the BC gelatinous membrane of the HSBC gel. The resulting HRP-immobilized silica particles (Si-HRPs) were encapsulated in HSBC gels using conventional methods [29]. The aqueous environment inside the HSBC gel helps prevent enzyme denaturation and maintain enzyme activity. Moreover, substrates of low molecular weight can react with the enzyme without interference from the BC gelatinous membrane. The activity of encapsulated HRP was evaluated using the TMB-H_2_O_2_ system. The dense cellulose nanofiber network prevents large-sized objects such as cells from penetrating into the HSBC gel’s hollow interior, therefore, enzyme-immobilized particles encapsulated in the HSBC gel are protected from immune cell attacks. Unlike conventional enzyme immobilization methods using hydrogels, the encapsulation of enzyme-immobilized particles using HSBC gel provides a new enzymatic reaction field with good biocompatibility, excellent strength, and a wide range of pH resistance.

## 2. Results and Discussion

### 2.1. Characterization of Si-HRPs

**Figure 1:**
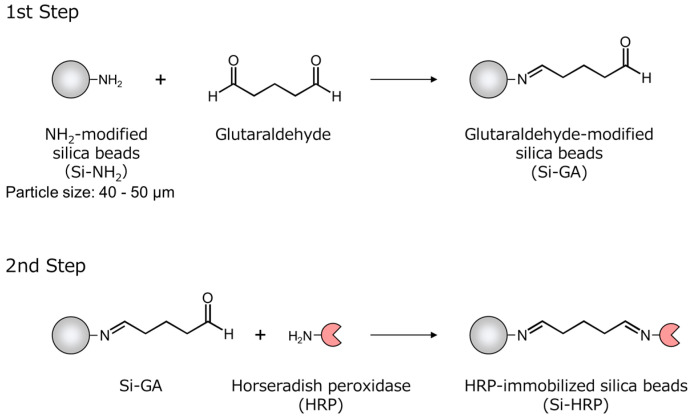
Schematic representation of the preparation of HRP-immobilized silica beads (Si-HRP).

Figure 1 shows the method of preparing the Si-HRPs. In the first step, immediately after the addition of glutaraldehyde, the formyl group of glutaraldehyde reacted with the amino group of NH_2_-modified silica bead (Si-NH_2_) particles to form a Schiff base, resulting in white Si-NH_2_ particles turning orange. In the second step, the obtained Si-HRPs turned red. The immobilized HRP on the Si-HRPs showed TMB oxidation (see Section 2.3), thus confirming the successful immobilization of HRP on glutaraldehyde-modified silica beads (Si-GAs). Table 1 indicates the results of the elemental analysis of Si-NH_2_, Si-GAs, and Si-HRPs. Significant weight loss at each step indicated the immobilization of glutaraldehyde and HRP. Weight loss was assumed to occur due to the combustion of organic components on the silica surface, and the amount of HRP on Si-HRPs was calculated using the ratio of organic components to residue (Organic/Residue (wt%)) according to Equations (1)–(4). The amount of immobilized HRP on Si-HRPs was calculated to be 17.2 µg/mg (0.0172 µg/µg).

(1) $$Weight\;loss\;\left(wt\%\right)=\left(\frac{\left(\left(Sample\;(\mu g)\right)-\left(Residue\;\left(\mu g\right)\right)\right)}{Sample\;\left(\mu g\right)}\right)\times 100=\left(\frac{Organic\;(\mu g)}{Sample\;\left(\mu g\right)}\right)\times 100$$

(2) $${\left(\frac{HRP}{Residue}\right)}_{Si-HRP}={\left(\frac{Organic}{Residue}\right)}_{Si-HRP}-{\left(\frac{Organic}{Residue}\right)}_{Si-GA}$$

(3) $$Amounts\;of\;HRP\;\left(\mu g\right)={\left(\frac{HRP}{Residue}\right)}_{Si-HRP}\times {\left(Residue\;\left(\mu g\right)\right)}_{Si-HRP}$$

(4) $$Immobilized\;HRP\;\left(\mu g/\mu g\right)=\frac{\left(Amounts\;of\;HRP\;\left(\mu g\right)\right)}{{\left(Residue\;\left(\mu g\right)+Organic\;\left(\mu g\right)\right)}_{Si-HRP}}$$

### 2.2. Preparation of HSBC Gels Encapsulating Si-HRPs

**Figure 2:**
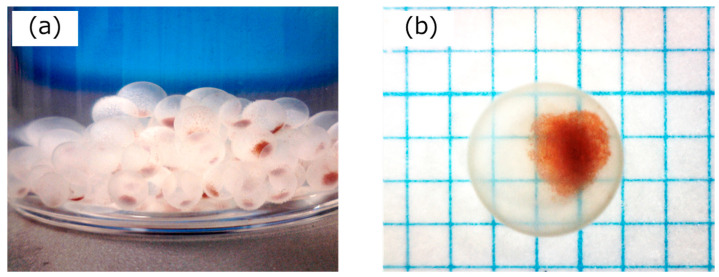
Photographs of the Si-HRP-encapsulated HSBC gels. (**a**) Si-HRP-encapsulated HSBC gel from a single experiment, with a yield of over 90% (>86 pieces). (**b**) An enlarged view of the Si-HRP encapsulated HSBC gel, showing reddish-brown Si-HRP particles.

Figure 2 shows photographs of the Si-HRP-encapsulated HSBC gels. The yield exceeded 90%, and our method reproducibly produced the Si-HRP-encapsulated HSBC gel (Figure 2a). This method formed a uniform BC gelatinous membrane (Figure 2b) on the surface of the Ca-Alg gel. This is consistent with our previous reports [28,29], demonstrating that when Ca-Alg gel dissolves in PBS, Si-HRPs precipitate within the HSBC gel and remain within the HSBC gel’s inner surface. The Si-HRP containing Ca-Alg gel was prepared by adding 20 µL of a Na-Alg solution containing 1.0 wt% Si-HRP, resulting in HSBC gel samples that each contained 200 µg of Si-HRP. Thus, using the value obtained in Equation 4 (0.00172 μg/μg), the HRP content within the HSBC gel was found to be 3.44 μg.

**Figure 3:**
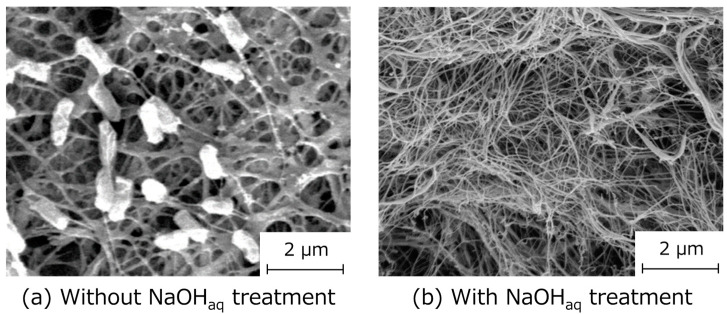
SEM images of BC gelatinous membrane of Si-HRP-encapsulated HSBC aerogels. (**a**) Non-treated sample, and (**b**) NaOH aqueous solution-treated sample.

SEM observations of the Si-HRP-encapsulated HSBC aerogels showed a network structure of cellulose nanofibers in the BC membrane (Figure 3). The presence of *K. xylinus* and cellular debris was confirmed as bacteriolysis treatment, and an NaOH aqueous solution was not performed in order to avoid deactivating the HRP (Figure 3a). SEM images of HSBC aerogels treated with the NaOH aqueous solution, which dissolved *K. xylinus* and cellular debris, demonstrated that the BC gelatinous membrane formed a network structure consisting of cellulose nanofibers with a diameter of about 30 nm. Importantly, the pore size of this network structure was less than 1 μm (Figure 3b), which was significantly smaller than the encapsulated Si-HRPs with a diameter of 40–50 µm. Consequently, these encapsulated Si-HRPs did not leak after being stored in Milli-Q water or acetate buffer solution for more than one month.

### 2.3. Activity Evaluation of Si-HRP-Encapsulated in HSBC Gels

**Figure 4:**
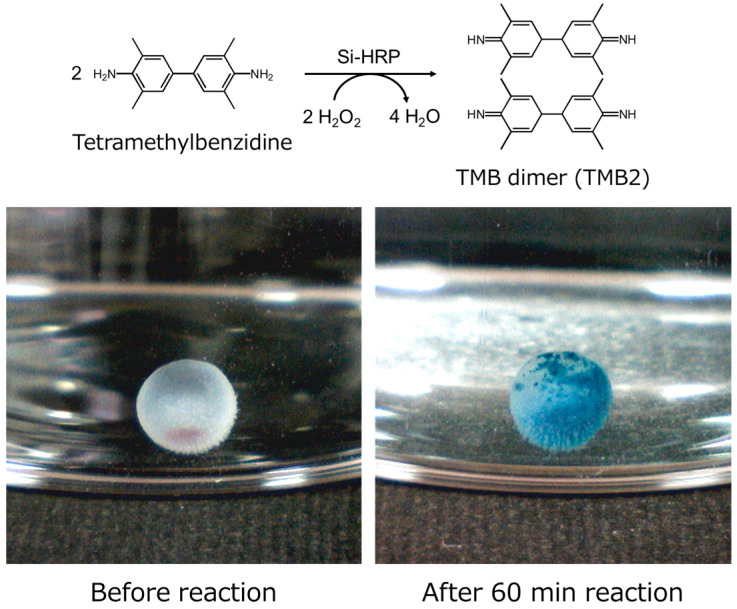
Photographs of Si-HRP-encapsulated HSBC gels after reaction with TMB.

When Si-HRPs encapsulated in HSBC gel were reacted with TMB substrate solution, the HSBC gel turned blue over time due to the formation of TMB dimers (TMB2), as shown in Figure 4. This result indicates that the encapsulated Si-HRPs utilized the internal space of the HSBC gel as a reaction field, generating TMB2 through substrate–enzyme reactions. This also suggests that encapsulation of Si-HRPs with HSBC gel does not deactivate HRP, and may similarly encapsulate other enzymes while maintaining their activity.

**Figure 5:**
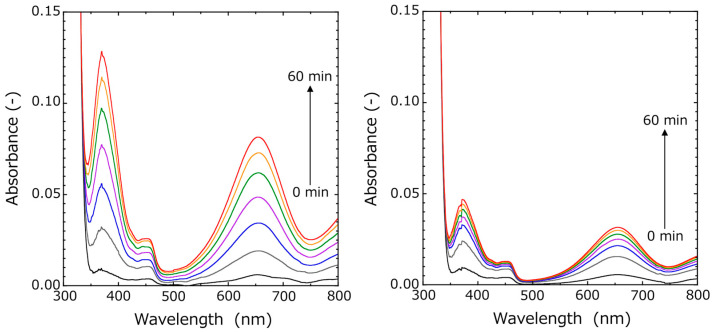
Time–dependent UV–Vis absorption spectra of TMB–H_2_O_2_ system in the presence of (**left**) Si-HRP encapsulated HSBC gel or (**right**) HSBC gel without Si-HRP.

The activity of Si-HRPs encapsulated in HSBC gels was evaluated in more detail using UV–Vis spectroscopy. Over time, both HSBC gels encapsulated with Si-HRP and HSBC gels without Si-HRP showed an increase in absorbance at 370 nm and 655 nm, derived from the formation of TMB2 (Figure 5). Gu et al. reported that TMB with H_2_O_2_ but without a catalyst undergoes slow oxidation [31]. The absorbance at 655 nm increased more significantly in the presence of Si-HRP-encapsulated HSBC gel (Figure 5 (left)).

**Figure 6:**
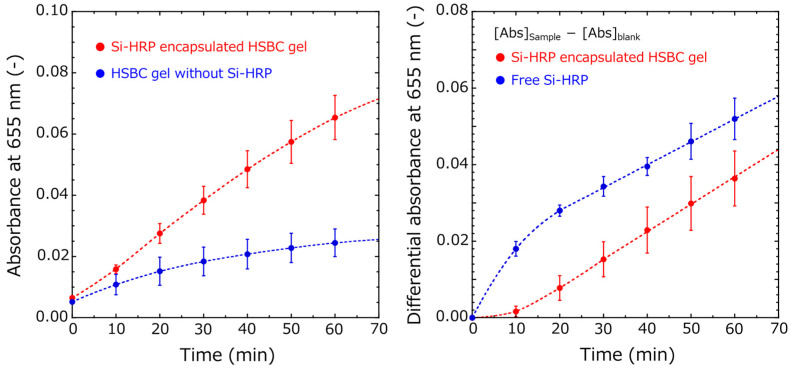
Evaluation of Si-HRP activity using UV–Vis spectra. (**Left**) Time-dependence of absorbance at 655 nm of Si-HRP-encapsulated HSBC gel and HSBC gel in TMB–H_2_O_2_ system. (**Right**) Time-dependence of the differential absorbance at 655 nm between samples and blanks in the TMB–H_2_O_2_ system.

The time-dependence of the difference in absorbance at 655 nm is shown in Figure 6 (left). While the absorbance increased in both HSBC gels with and without Si-HRP, the increase was greater for the Si-HRP-encapsulated HSBC gel. The differential absorbance was then used to evaluate the activity of Si-HRPs. The differential absorbance was calculated by subtracting the absorbance of the HSBC gel without Si-HRP (blank) from that of the Si-HRP-encapsulated HSBC gel. For comparison, the differential absorbance was also calculated by subtracting the absorbance of the TMB solution from that of the unencapsulated Si-HRP. The time dependence of the differential absorbance is shown in Figure 6 (right). In the Si-HRP encapsulated HSBC gel, there was an initial lag phase (induction phase), where the absorbance of TMB dimers ([Abs]_655 nm_) was barely detectable until 10 min, followed by a linear increase afterward. Conversely, the activity of unencapsulated Si-HRP showed a continuous increase in [Abs]_655 nm_ from the start of the reaction, with a constant increase rate observed after 20 min. The slope after 20 min was almost equal to 7.18 ± 1.04 × 10^−4^ for Si-HRP encapsulated HSBC gel and 6.55 ± 0.77 × 10^−4^ for free Si-HRP, indicating that immobilized HRP on the silica particles was not deactivated by the encapsulation process.

(Red circle) [Abs]_Si-HRP encapsulated HSBC gel_ − [Abs]_HSBC gel_, (Blue circle) [Abs]_Free Si-HRP_ − [Abs]_TMB solution_

Furthermore, this fact indicated that the presence of *K. xylinus* residues in the BC gelatinous membrane comprising HSBC gel does not affect the activity of HRP. During the cultivation process, Si-HRP is included within the alginate gel, protecting it from inactivation by *K. xylinus* on the surface of the alginate gel. Therefore, *K. xylinus* residues were considered to affect the permeability of TMB and TMB2 through the BC gelatinous membrane. Many researchers have proposed models for solute diffusion in hydrogels [32,33,34]. Clague and Phillips employed a combined hydrodynamic/obstruction simulation model to study solute diffusion within a random network of cylindrical fibers [35]. Their expression for the reduction in solute diffusivity is as follows:

(5) $$\frac{D_{g}}{D_{0}}={\left(1+\frac{2}{3}\alpha \right)}^{-1}exp\left(-\pi \varphi^{0.174\ln\left(59.6\frac{r_{f}}{r_{s}}\right)}\right)$$

where

(6) $$\alpha =\varphi {\left(\frac{r_{s}+r_{f}}{r_{f}}\right)}^{2}$$

D_g_ is solute diffusion in a gel, D_0_ is the diffusion coefficient of the solute in the liquid at infinite dilution, φ is the volume fraction of polymer in the gel, r_f_ is the radius of the polymer fiber, and r_s_ is the radius of the solute. These results demonstrated that when the r_f_/r_s_ ratio is large or the volume fraction is small, D_g_/D_0_ ≒ 1.0, there is no effect on solute diffusion. The hydrodynamic radius of TMB2 was 0.55 nm (see Table 2), and the diameter of the cellulose nanofibers was 30 nm (see Section 2.2), resulting in an r_f_/r_s_ ratio of 27.3. The porosity of BC gel was greater than 99%, and the volume fraction of cellulose nanofibers was less than 1% [36]. At r_f_/r_s_ = 27.3 and a cellulose nanofiber volume fraction of 1%, D_g_/D_0_ = 0.984. This indicated that the diffusion of TMB2 and smaller TMBs in the BC gelatinous membrane were nearly unaffected. Since *K. xylynus* (approximately 1 μm) were fewer in number, their volume fraction was smaller, resulting in a larger r_f_/r_s_ value. Therefore, it is suggested that the *K. xylynus* residues do not affect the diffusion of TMB2 and TMB in the BC gel membrane.

### 2.4. Discussion

**Figure 7:**
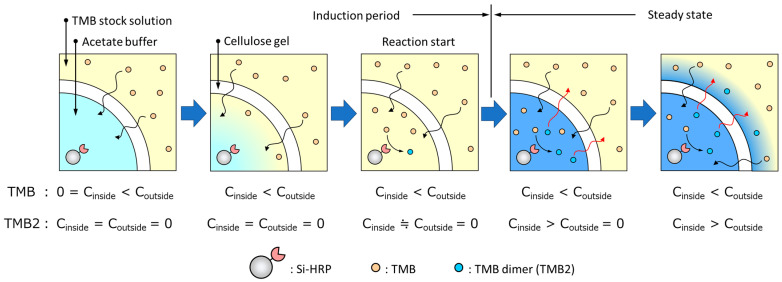
Schematic diagram showing the steady state release of TMB2.

The induction phase of [Abs]_655 nm_ in the Si-HRP-encapsulated HSBC gel, as shown in Figure 6 (right), is likely due to the following process (Figure 7). Firstly, the TMB substrate permeates into the interior of the HSBC gel through the BC gelatinous membrane. Initially, the TMB concentration inside the HSBC gel is zero, but it is high outside, hence, TMB diffuses and penetrates spontaneously into the HSBC gel. Secondly, the permeated TMB is oxidized with H_2_O_2_ by Si-HRP to form TMB2. Thirdly, as TMB2 accumulates inside the gel, it spontaneously permeates through the BC gel membrane and diffuses outward. These three processes—substrate permeation, enzyme reaction, and TMB2 release—are suggested to be contributing factors to the induction period.

After the initial induction period, the rate of increase in [Abs]_655 nm_ remained constant, suggesting that TMB2 release was steady. As TMB2 is generated inside the HSBC gel, the TMB concentration inside the HSBC gel decreases, prompting TMB to be supplied from the outside. This continuous supply maintains a constant TMB concentration within the HSBC gel. With this constant TMB concentration, TMB2 is presumed to be generated at a consistent rate. The generated TMB2 permeates the BC gelatinous membrane of HSBC gel and is released outside. The driving force for this TMB2 release is the concentration difference inside and outside the HSBC gel, which follows diffusion according to Fick’s law.

## 3. Conclusions

In this study, we successfully encapsulated enzyme-immobilized silica particles (Si-HRPs) using HSBC gels composed of cellulose nanofibers. These nature-based materials exhibit excellent mechanical properties, good biocompatibility, and stability across a broad pH range [30]. Since this encapsulation process can be performed below 40 °C, it was successful without deactivating the immobilized HRPs. The encapsulated Si-HRP effectively oxidized TMB, albeit with an observed induction period in the reaction’s progress. This induction period arose from three steps: the penetration of TMB into the HSBC gel through the BC gelatinous membrane, the oxidation of TMB at the Si-HRP surface, and the release of generated TMB2 outside the HSBC gel. Subsequently, the concentration of TMB2 inside the HSBC gel was maintained by the continuous supply and reaction of TMB, ensuring a constant release rate of TMB2 from the HSBC gel. These findings suggest that HSBC gels composed of cellulose nanofibers are suitable materials for the encapsulation of biological components such as enzyme catalysts.

The activity of the encapsulated Si-HRPs was detected using the TMB-H_2_O_2_ system, indicating that this method can encapsulate the enzyme without deactivation. Since HSBC gels are formed by a network of biocompatible cellulose nanofibers, immune cells such as leukocytes cannot enter the hollow interior, thus, the enzyme-immobilized particles encapsulated inside the HSBC gel are protected from immune cell attacks. Therefore, the encapsulation technique presented in this study is expected to facilitate the delivery of enzymes and catalysts that are not originally present in the in vivo environment.

## 4. Materials and Methods

### 4.1. Materials

The Hestrine–Schramm medium (HS medium) [38] was used for incubating the bacterial strain. The standard HS medium consisted of a mixture of 3.0 g D-glucose (Kanto Chemical Co., Inc., Chuo-ku, Tokyo, Japan)), 0.5 g mannitol (Kanto Chemical Co., Inc.), 0.5 g peptone (HIPOLYPEPTONE^TM^, Nihon Pharmaceutical Co. Ltd., Izumisano City, Osaka, Japan), 0.5 g Bacto^TM^ yeast extract (BD Biosciences, Franklin Lakes, NJ, USA), and 0.1 g magnesium sulfate heptahydrate (MgSO_4_∙7H_2_O: Kanto Chemical Co., Inc.) in 100 mL Milli-Q water. The density of the HS medium at 30 °C was measured as 1.02 g/cm^3^ using a Baume hydrometer. Silicone oils (KF-56A: 0.995 g/cm^3^, 15 mm/s^2^, ethanol-soluble oil) were obtained from Shin-Etsu Chemical Co., Ltd. (Chiyoda-ku, Tokyo, Japan). Sodium alginate, calcium chloride, dimethyl sulfoxide, hydrogen peroxide (35% in water), and NH_2_-modified silica gel (Silica Gel 60 (spherical) NH_2_, diameter: 40~50 µm) were purchased from Kanto Chemical Co., Inc. and used as received. Glutaraldehyde (50% in water), horseradish peroxidase (HRP), and *N*, *N*, *N’*, *N’*-Tetramethylbenzidine (TMB) were purchased from Tokyo Chemical Industry Co., Ltd. (Chuo-ku, Tokyo, Japan) and used as received. Acetate buffer solution (0.1 mol/L, pH 5.0) was purchased from Nacalai Tesque, Inc. (Nakagyou-ku, Kyoto, Japan) and used as received.

### 4.2. Preparation of HRP-Immobilized Silica Beads

HRP-immobilized silica beads were prepared using the glutaraldehyde bridging method [39,40,41]. Glutaraldehyde (50% in water, 0.8 mL) was added to 20 mL of water suspending 100 mg of NH_2_-modified silica beads (Si-NH_2_); this suspension was stirred for 1 h at room temperature (Figure 1, 1st step). After washing with water, the glutaraldehyde-modified silica beads (Si-GA) were suspended in 8 mL of 0.1 mol/L acetate buffer (pH 5.0). Then, a 1.0 mg/mL HRP solution (16 mL, acetate buffer (pH 5.0)) was added to this suspension and stirred for 1 h at room temperature (Figure 1, 2nd step). The HRP-immobilized silica beads (Si-HRPs) were washed thoroughly with Milli-Q water and stored at 4 °C. Organic components were determined using an elemental analyzer with a sample furnace temperature of 950 °C (MICRO CORDER JM10, J-SCIENCE LAB Co., Ltd., Minami-ku, Kyoto, Japan). In addition, the residue weight was measured using a microgram balance (Sartorius, MSE3.6P000DM, readability: 1 μg). Then, the amount of immobilized HRP was calculated from the organic components and weight loss (%) of Si-NH_2_, Si-GA and Si-HRP (see Section 2.1).

### 4.3. Preparation of Si-HRP-Encapsulated Hollow-Type Spherical BC Gels

**Figure 8:**
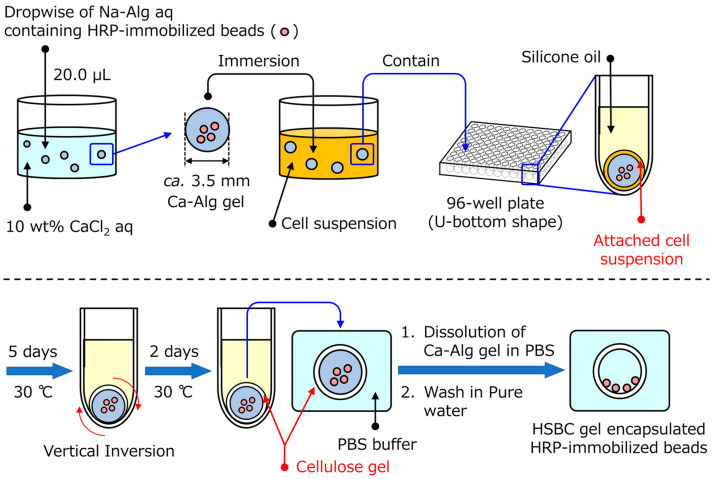
A schematic of the production process of the hollow-type spherical bacterial cellulose (HSBC) gel encapsulating HRP-immobilized silica particles (Si-HRPs).

Figure 8 shows the methods of preparing the Si-HRP-encapsulated HSBC gels. Spherical alginate gels (Ca-Alg gel), including Si-HRP, were prepared by dropping 20.0 µL of a 1 wt% sodium alginate aqueous solution (Na-Alg aq) suspending 1 wt% of Si-HRP into a 10 wt% calcium chloride aqueous solution (CaCl_2_ aq). The obtained Ca-Alg gels were washed via immersion in Milli-Q water.

The HS medium was sterilized via autoclaving, and then *Komagataeibacter xylinus* (IFO13772, synonym: *Gluconacetobacter xylinus*) was cultured in the HS medium at 30 °C for 3 days. The spherical Ca-Alg gels, which included SI-HRPs, were immersed in the cultured cell suspension and inoculated with *K. xylinus* for one day at 30 °C. Spherical Ca-Alg gels, in which the cell suspension remained at the Ca-Alg gel surface, were immersed in each well of a U-shaped bottom 96-well plate filled with silicone oil. While maintaining these states, the *K. xylinus* in the cell suspension was cultured for a specified period at 30 °C. Using this method, the BC gelatinous membrane was biosynthesized by *K. xylinus* at the interface between cell suspension attached to the alginate gel and silicone oil. To create a uniform BC gel membrane, the gel was inverted vertically on day 5 of incubation, and then incubated for an additional 2 days, for a total of 7 days. After the BC gelatinous membrane was biosynthesized, an alginate gel was dissolved in a phosphate-buffered solution to prepare an HSBC gel with Si-HRP.

The yield of the Si-HRP-encapsulated HSBC gel was calculated using the following Equation (7).

(7) $$Yield\;(\%)=\frac{Number\;of\;obtained\;Si-HRP\;encapsulated\;HSBC\;gel}{Number\;of\;Ca-Alg\;gel\;used\;in\;cultivation}\times 100$$

### 4.4. Preparation of HSBC Aerogel Using Supercritical CO_2_

Water-swollen HSBC gel was placed in a large quantity of ethanol and washed thoroughly, and the swelling solvent completely changed from water to ethanol. The gel was dried using a supercritical CO_2_ (scCO_2_) technique without disintegrating its microstructure [27,28,29,42]. The drying process was conducted under conditions of 60 °C, 20 MPa, a CO_2_ flow rate of 2.0 mL/min, and a duration of 5 h. The drying apparatus consisted of a CO_2_ delivery pump (SCF-Get, JASCO Corporation, Hachioji, Tokyo, Japan), a 50 mL pressure vessel, a gas pressure regulator (SCF-Bpg, JASCO Corporation, Hachioji, Tokyo, Japan), and a constant-temperature water bath (BK33, Yamato Scientific Co. Ltd., Chuo-ku, Tokyo, Japan).

### 4.5. Microstructure Measurements of HSBC Gels

The microstructure of the HSBC aerogels was observed using a field-emission scanning electron microscope (FE-SEM: Hitachi High-Technologies Corporation S-4500, Minato-ku, Tokyo, Japan) with an acceleration voltage of 10 kV. For the pretreatment prior to FE-SEM observation, the deposition of Pt-Pd was performed via ion sputtering (Hitachi High-Technologies Corporation E-1010, Minato-ku, Tokyo, Japan).

### 4.6. Evaluation of HRP Activity

The assay conditions were essentially those described by Bos et al. [43]. Solution A was prepared by dissolving 30.3 mg of TMB in 6 mL of DMSO. A TMB stock solution was prepared by mixing 100 µL of Solution A with 9.9 mL of acetate buffer (pH 5.0) containing 1.0 µL of H_2_O_2_. The Si-HRP-encapsulated HSBC gel was placed in 3 mL of TMB stock solution in a square quartz cell (optical path length of 1 cm), and the reaction was conducted at 37 °C with a stirring speed of 100 rpm. Enzyme activity was evaluated by measuring the spectra every 10 min using a UV–Vis spectrometer (JASCO Corporation, V-530, Hachioji, Tokyo, Japan).

For comparison, the activity of unencapsulated Si-HRP was similarly evaluated under ten-fold higher feeding conditions (Si-HRP: 2.0 mg, TMB stock solution: 30.0 mL). Enzyme activity was evaluated using UV–Vis spectrophotometry of the supernatant solution after centrifugation (5000 rpm, 30 s) of 3.0 mL solutions collected at 10-min intervals.

## Figures and Tables

**Table 1:** Results of elemental analysis of Si-NH_2_, Si-GAs, and Si-HRPs.

Sample Name	Weight Loss (wt%)	Organic/Residue (wt%)
Si-NH_2_	18.0 ± 0.1	21.9 ± 0.1
Si-GA	31.5 ± 0.5	46.1 ± 0.8
Si-HRP	32.7 ± 0.2	48.6 ± 0.3

**Table 2:** Hydrodynamic radius and diffusion coefficient of FITC–Dextran and TMB2.

Sample Name	Molecular Weight (g/mol)	Temp. (°C)	Hydrodynamic Radius (nm)	Diffusion Coefficient * (m^2^/s)
FITC-Dextran	1.0 × 10^4^	25.0	2.3 [37]	1.1 × 10^−10^
FITC-Dextran	1.0 × 10^4^	37.0	2.3 [37]	1.4 × 10^−10^
TMB2	472.63	37.0	0.55 **	5.9 × 10^−10^

* The diffusion coefficient was calculated using the Einstein–Stokes equation. ** The molecular radius, r, of TMB2 that was assumed to be a sphere was calculated using the following equation: $\frac{4}{3}\pi r^{3}=\frac{M_{w}}{d\cdot N_{A}}$, where M_w_ is the molecular weight, d is density, and N_A_ is Avogadro’s constant.

## Data Availability

Data are contained within the article.
